# Unraveled: Coil Migration After Embolization of Gastric Varices

**DOI:** 10.14309/crj.0000000000001748

**Published:** 2025-06-27

**Authors:** Sheel Patel, Dylan Flaherty, Nana Baah, Endashaw Omer

**Affiliations:** 1 Division of General Internal Medicine, Department of Medicine, University of Louisville, Louisville, KY; 2 Division of Gastroenterology, Hepatology, and Nutrition, Department of Medicine, University of Louisville, Louisville, KY; 3 Division of Interventional Radiology, Department of Radiology, University of Louisville, Louisville, KY

**Keywords:** coil migration, gastric varix, embolization, transjugular intrahepatic portosystemic shunt, endoscopy

## Abstract

Gastric varices are a severe complication of portal hypertension and may lead to life-threatening bleeding. Endovascular interventions, including transjugular intrahepatic portosystemic shunt and coil embolization, are effective treatment strategies. However, coil migration is a rare but serious complication. We present a unique case of coil migration from a gastric varix to the duodenum after transjugular intrahepatic portosystemic shunt and embolization.

## INTRODUCTION

Gastric varices (GOV2) are dilated submucosal veins in the stomach that arise as part of a complex network of vascular shunts between the portal and systemic circulation.^1^ They typically develop in the setting of portal hypertension, with or without cirrhosis. The incidence of bleeding from gastric varices is reported to be between 16% and 45% over a 3-year period.^2^ Although less common than bleeding from esophageal varices, gastric variceal bleeding is more severe and often requires more blood transfusions.^3^ Treatment options for gastric variceal bleeding include pharmacological therapy, endoscopic cyanoacrylate glue injection, endoscopic variceal ligation, and endovascular intervention.^4^ In patients where bleeding continues to happen despite initial temporizing measures, endovascular interventions such as transjugular intrahepatic portosystemic shunt (TIPS) or coil embolization can be used. Coil migration is an extremely rare complication, and it can lead to serious consequences based on where the coil migrates. We present a case of coil migration from a GOV2 to the duodenum after coil embolization of a GOV2 and TIPS 3 years ago. To our knowledge, this is the first reported case where the coil remained embedded in the GOV2 but unraveled into the duodenum.

## CASE REPORT

**Figure 1.**
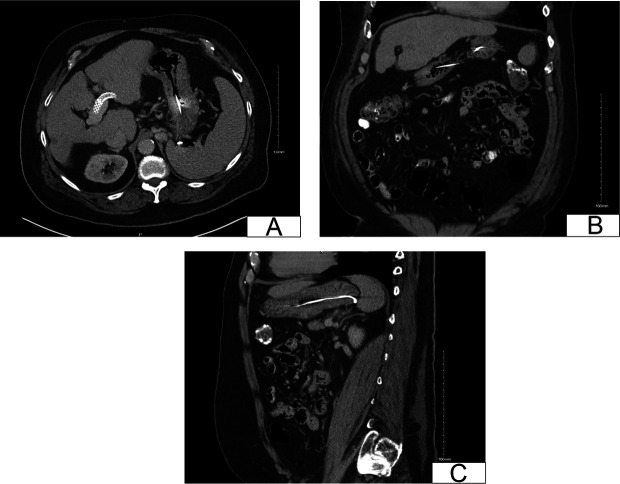
Computed tomography scan (CT) of the abdomen/pelvis showing a thin-metallic object within the duodenum and stomach. Transverse view (A), coronal view (B), and sagittal view (C).

**Figure 2.**
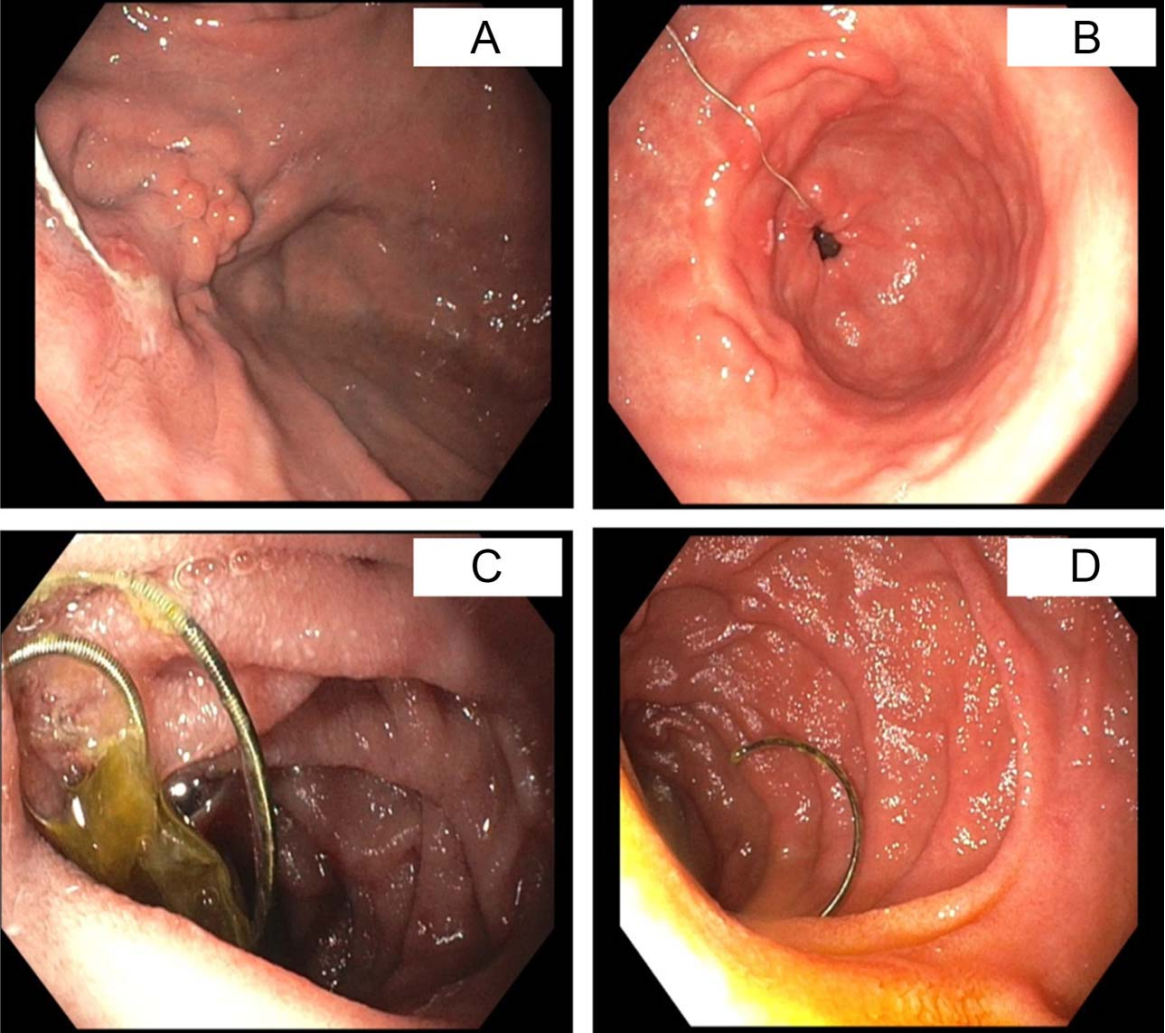
Coil embedded in a previous gastric varix (A). Coil traversing through the gastric antrum (B). The coil moves through the small intestine (C). The distal end of the coil ends in the second portion of the duodenum (D).

A 66-year-old man with a history of hepatocellular carcinoma and liver cirrhosis presented to his primary care office with abdominal pain and nausea. An abdominal x-ray and follow-up computed tomography of the abdomen and pelvis revealed a thin, wire-like metallic density, likely located within the stomach and duodenum (Figure 1). Three years earlier, he was hospitalized for gastric variceal bleeding and underwent TIPS and coil embolization under fluoroscopic guidance. Two standard detachable RubyCoils (40 mm × 60 cm and 36 mm × 60 cm) and 3 PackingCoils (45 and 60 cm) were used (Penumbra, Alameda, CA). No gelatin or cyanoacrylate was used. The patient denied any history of foreign body ingestion before the procedure. To further investigate the metallic density, an esophagogastroduodenoscopy was performed, which showed an unraveled coil from his prior embolization. While the coil was still present in the GOV2, it had unraveled to the third portion of the duodenum (Figure 2). Endoscopic removal was not attempted as the coil was still in the decompressed varix (Figure 2). Interventional radiology (IR) was called to bedside to assess if removal was possible. However, given that the patient did not experience any symptoms and the potential risk of bleeding, the coil was left in place. He was advised to return if he developed any symptoms related to the unraveled coil. If needed, removal would be planned in coordination with IR.

## DISCUSSION

Coil migration after TIPS and embolization is a rare but potentially serious complication. The precise mechanism is not well defined but may involve persistent high-pressure portal flow, anatomical factors such as variceal proximity to the gastroesophageal junction, technical issues including inadequate coil deployment, or insufficient use of stabilizing agents such as cyanoacrylate glue.^4–6^ In our case, packing density was suboptimal as no cyanoacrylate or gelatin was used, which may have failed to establish a stable scaffold, predisposing to delayed unraveling and migration.^7^ While endovascular techniques such as TIPS and coil embolization are highly effective in controlling gastric variceal bleeding, complications such as migration pose significant clinical challenges due to the potential for bleeding, perforation, or distant embolization.

A review of previously reported cases highlights the broad variability in timing, location, and clinical sequelae associated with coil migration after embolization of gastric varices (Table 1). Migration has been reported from as early as 24 hours to over a year following the procedure, with involved sites ranging from the gastric lumen to the pulmonary artery and cardiac chambers. Clinical outcomes also vary widely, from asymptomatic findings to life-threatening events such as massive gastrointestinal bleeding. Three years after embolization, our patient was incidentally found to have a coil that had unraveled into the third portion of the duodenum, while its proximal loops remained embedded within a decompressed GOV2. To our knowledge, this is the first reported case of asymptomatic, partial coil migration into the duodenum occurring over such an extended period, highlighting its uniqueness and educational significance.

**Table 1.** Reported cases of coil migration after TIPS or embolization for gastric varices

Author/year/journal	Procedure performed	Time to detection	Organ reached/sequelae	Management
Current case	TIPS + coil embolization	3 yrs	Duodenum (D3)/asymptomatic	Conservative
Kupkova et al., 2006 (*Folia Gastroenterology et Hepatology*)^10^	TIPS + coil embolization	3 wk	Gastric lumen/hematemesis	Attempted endoscopic removal; conservative after failure
Zhang et al., 2022 (*Journal of Cardiothoracic Surgery*)^11^	TIPS + coil embolization with glue^a^	∼1 yr	Gastric lumen/asymptomatic	Attempted endoscopic removal; conservative after failure
Sandri et al., 2007 (*Journal of Vascular and Interventional Radiology*)^12^	TIPS + coil embolization	7 mo	Stomach fundus/hematemesis	Surgical removal (partial gastrectomy); patient died
Tepox-Padron et al., 2024 (*Journal of Gastrointestinal Endoscopy*)^13^	EUS-guided coil embolization with glue^b^	24 h	Pulmonary artery/hypoxia	Conservative
Pusateri et al., 2020 (American College of Gastroenterology Case Reports Journal)^14^	TIPS + balloon-occluded antegrade transvenous obliteration (BATO)	∼1 yr	Fundus of stomach/hematemesis	Conservative
Hussain and Ghaoui, 2011 (*Journal of Interventional Gastroenterology*)^15^	TIPS + coil embolization	2 wk	Stomach fundus/asymptomatic	Conservative
Rowley and Suarez, 2021 (*Journal of Gastrointestinal Endoscopy*)^16^	EUS-guided coil embolization with glue^c^	2 wk	Right ventricle/pleural effusion	Repeat coil embolization; initial coil left in place

Reported cases of coil migration after embolization for gastric varices, including the procedure performed, time to detection, organ involved, clinical sequelae, and management strategy.

TIPS, transjugular intrahepatic portosystemic shunt.

a NBCA is only used.

b n-butyl-2-cyanoacrylate and lipiodol (NBCA) used.

c Dermabond used.

Management strategies in prior reports have ranged from conservative monitoring to endoscopic or surgical retrieval. In asymptomatic patients with high bleeding risk, conservative management is typically preferred. However, retrieval has been successfully achieved in select cases. For example, endoscopic removal has been reported in settings such as splenic artery and super mesenteric artery pseudoaneurysm embolization, using endoscopic scissors and/or hot biopsy forceps to cut and remove the coils.^8,9^ Conversely, Kupkova et al and Zhang et al illustrate failed endoscopic attempts within the gastric lumen using forceps/cold biopsy snares, ultimately requiring conservative management.^10,11^ Surgical interventions such as partial gastrectomy have also been used in more severe presentations, though associated morbidity and mortality can be significant.^12^

Given the potential for delayed complications such as mucosal ulceration or perforation, we recommend a structured surveillance plan for patients with retained or migrated coils. Scheduled follow-up with esophagogastroduodenoscopy and/or computed tomography scan at 6 months and then annually may allow early detection of progressive migration or erosion.^14^ Patients should be counseled to seek prompt evaluation if they experience new abdominal pain, gastrointestinal bleeding, chest pain, trouble breathing, or signs of infection. If our patient develops symptoms in the future, endoscopic retrieval would be attempted using polypectomy snare, hot biopsy forceps, or endoscopic scissors if needed to cut or ablate exposed wire ends. If unsuccessful or the bleeding risk is too high, IR-guided retrieval or surgical intervention may be considered, depending on clinical stability and anatomical location.

This case highlights the importance of long-term vigilance following variceal embolization and contributes to the limited but growing body of literature on coil migration. As more cases are documented, evidence-based guidelines for the surveillance and management of this rare complication can be developed.

## DISCLOSURES

Author contributions: Concept and design: S. Patel and E. Omer. Experiments and procedures: E. Omer. Data interpretation: S. Patel, D. Flaherty, N. Baah, and E. Omer. Writing the article draft: S. Patel and D. Flaherty wrote the first draft. All the authors reviewed, provided intellectual input, and approved the final draft. E. Omer is the article guarantor.

Financial disclosure: None to report.

Informed consent was obtained for this case report.

## ABBREVIATIONS:

CT: computed tomography
ECI: endoscopic cyanoacrylate injection
EGD: esophagogastroduodenoscopy
EVL: endoscopic variceal ligation
GOV2: gastroesophageal varices type 2
IR: interventional radiology
NBCA: N-butyl-2-cyanoacrylate
TIPS: transjugular intrahepatic portosystemic shunt
